# The Preliminary Study on the Proapoptotic Effect of Reduced Graphene Oxide in Breast Cancer Cell Lines

**DOI:** 10.3390/ijms222212593

**Published:** 2021-11-22

**Authors:** Rafał Krętowski, Agata Jabłońska-Trypuć, Marzanna Cechowska-Pasko

**Affiliations:** 1 Department of Pharmaceutical Biochemistry, Medical University of Bialystok, 15-089 Bialystok, Poland; mapasko@gmail.com; 2 Division of Chemistry, Biology and Biotechnology, Bialystok University of Technology, 15-351 Bialystok, Poland; a.jablonska@pb.edu.pl

**Keywords:** apoptosis, breast cancer, oxidative stress, proliferation, rGO

## Abstract

Breast cancer is the most common cancer diagnosed in women, however traditional therapies have several side effects. This has led to an urgent need to explore novel drug approaches to treatment strategies such as graphene-based nanomaterials such as reduced graphene oxide (rGO). It was noticed as a potential drug due to its target selectivity, easy functionalisation, chemisensitisation, and high drug-loading capacity. rGO is widely used in many fields, including biological and biomedical, due to its unique physicochemical properties. However, the possible mechanisms of rGO toxicity remain unclear. In this paper, we present findings on the cytotoxic and antiproliferative effects of rGO and its ability to induce oxidative stress and apoptosis of breast cancer cell lines. We indicate that rGO induced time- and dose-dependent cytotoxicity in MDA-MB-231 and ZR-75-1 cell lines, but not in T-47D, MCF-7, Hs 578T cell lines. In rGO-treated MDA-MB-231 and ZR-75-1 cell lines, we noticed increased induction of apoptosis and necrosis. In addition, rGO has been found to cause oxidative stress, reduce proliferation, and induce structural changes in breast cancer cells. Taken together, these studies provide new insight into the mechanism of oxidative stress and apoptosis in breast cancer cells.

## 1. Introduction

Breast cancer is one of the most frequently diagnosed neoplasms in women [1]. According to the American Cancer Society, in 2019, 268,000 new cases in women were diagnosed worldwide [2]. Until now, chemotherapy and radiotherapy have been the most frequent therapeutic options for the treatment of breast cancer. Unfortunately, some normal cells are also damaged by these methods. An important area in cancer research is the search for new compounds with high toxicity and selectivity in killing cancer cells. Due to their low toxicity in animal models, some graphene nanomaterials have been used as alternative treatments for breast cancer [3].

Graphene (G) and its derivatives, referred to as nanomaterials from the graphene family (GFNs), include graphene oxide (GO), its reduced form (rGO), single- or few-layer graphene, graphene nanosheets (GNS), and graphene nanoribbons [4,5]. However, the most important chemical derivative of graphene is GO. It can consist of hydroxyl, epoxide and carboxyl functional groups on its base surface and flat edges and is typically one to several hundred nanometers in size and 1–2 nm thick (1–3 layers). Through the reduction reaction, rGO can be synthesized by removing the oxygen-containing functional groups. GO has been used in several biomedical applications because of its excellent electrical and electronic, energetic, thermal, mechanical, optical, and biological properties. There are also biomedical applications of GO and rGO, including bio-imaging, drug delivery, antimicrobial effects or as a biosensor [6,7]. rGO, which includes graphene nanomaterials, may have potential applications in the treatment of cancer [6]. Hatama at al. demonstrated that curcumin-functionalized rGO sheets with concentrations < 70 μg/mL showed no significant changes in cytotoxicity and cell morphology. Meanwhile, they can induce further growth of cancer cells. At concentrations ≥ 70 μg/mL, some cytotoxic effects inducing slight cell death, cell apoptosis and cell morphological changes were observed [7]. Gnanasakar et al. demonstrated a significant cytotoxic effect of prepared nanocomposites against MDA-MB-468 and MDA-MB-231 cell lines compared to normal cells by inducing apoptosis. The overall result of this study suggests that the prepared metal-rGO nanocomposites functionalized with 5,7-dihydroxy flavones have a bright future for cancer chemotherapeutic purposes [8].

To date, there are no clear mechanisms by which rGO induces cytotoxicity. Various physiochemical properties of rGO, including size, shape, structure, hydrophobicity, surface functionalization, dose administration, purity and elemental composition, allow rGO to exhibit many mechanisms of action in cancer cells [9,10,11]. Key mechanisms that appear to be related to GFNs include the production of reactive oxygen species (ROS). The oxidative stress observed after the administration of rGO is usually caused by a decrease in the main antioxidant glutathione (the GSH-reduced form) and a strong increase in the ROS level [11].

Oxidative stress is a condition in the cell caused mainly by the presence of compounds that act as inducers of oxidative damage. Among these compounds, lipid peroxides, oxidized proteins and oxidized sugars may be mentioned. The main cellular macromolecules, which include structural and enzymatic proteins and DNA, are very sensitive to oxidative damage. Oxidative stress markers used to analyze oxidative damage to cells include protein oxidation, lipid peroxidation, and oxidation of DNA. Protein oxidation can result in disruption of the polypeptide chain, cross-linking in the polypeptide chains, and modification of amino acid residues. This can lead to a modification or even complete loss of the biological activity of the protein. The SH content of proteins as a marker of oxidative changes is in balance with the reduced form of glutathione (GSH), which plays an important role in protecting cells from damage caused by free radicals, peroxides and other compounds. Another process that occurs as a result of action and with the participation of free radicals is lipid peroxidation. It consists in the oxidation of unsaturated fatty acids present in the lipids of the cytoplasmic membranes. As a result, peroxide compounds and other free radicals are formed, which initiate further peroxidation reactions [12].

Apoptosis plays a pivotal role in the control of tumor growth and is a tightly controlled process that maintains tissue homeostasis. Apoptosis is characterized by morphological changes such as cell shrinkage, nuclear condensation and fragmentation, and the appearance of apoptotic bodies. Furthermore, this process can be activated through several signalling pathways, including the death receptor (extrinsic) and mitochondrial (intrinsic) pathways. The extrinsic pathway is mediated by the tumor necrosis factor receptor (TNF-R) superfamily, when the receptors are bound by the ligands Fas ligand, tumor necrosis factor (TNF) and TNF-related apoptosis-inducing ligand (TRAIL). The intrinsic pathway is under the control of mitochondrial proenzymes with various functions, including disturbance of the mitochondrial membrane potential (MMP) and the release of proapoptotic factors into the cytosol as well as a second mitochondria-derived activation of caspases, (Figure S1) [4,13].

Recent research has demonstrated that rGO can induce size- and dose-dependent cytotoxicity in different human cell lines, both normal and cancerous, although the mechanism of action is unclear [14]. Chatterjee et al. reported that GO induced a dose-dependent cytotoxicity and apoptosis, indicating damage to the plasma membrane by loss of the structural integrity of the plasma membrane. This phenomenon was associated with the strong physical interaction of graphene with the phospholipid bilayer [13]. Li et al. indicated that exposure of a lung cancer cell line (GLC-82) to GO induced the redistribution of cytoplasmic lactate dehydrogenase (LDH), which additionally suggests a loss of plasma membrane integrity [15]. Jaworski et al. presented microscopic visualisations of interactions between graphene and glioblastoma cell lines [9]. They showed that graphene platelets adherent to the cell body, causing mitochondrial disruption and apoptosis by generating oxidative stress [16].

The aim of current study was to evaluate the effect of in vitro rGO on cytotoxicity, morphology, proliferation, oxidative stress and apoptosis in breast cancer cell lines. In our study, we used five different breast cancer cell lines. These cells are characterized by various morphology, size, apoptosis-related gene activity, cell cycle regulation, and expression of the estrogen receptor. We were the first to study the cytotoxicity of rGO on the MDA-MB-231, Hs 578T, T47D, MCF-7 and ZR-75-1 cell lines.

## 2. Results

### 2.1. The Effect of rGO on Viability and Proliferation

**Figure 1:**
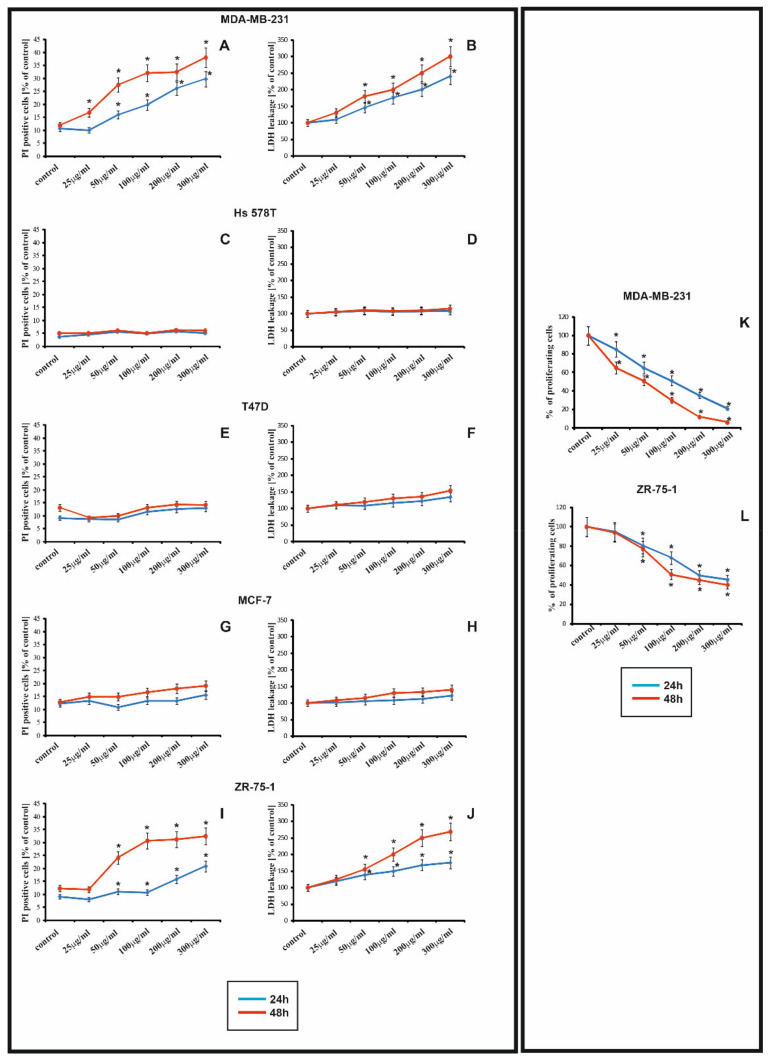
The effect of rGO cytotoxicity and proliferation on T-47D, MCF-7, ZR-75-1, MDA-MB-231, Hs 578T cell lines, was determined using LDH (**B**,**D**,**F**,**H**,**J**), PI (**A**,**C**,**G**,**E**,**I**) and crystal violet (**K**,**L**) test. Viability of MDA-MB-231 (**A**,**B**), Hs 578T (**C**,**D**), T-47D (**E**,**F**), MCF-7 (**G**,**H**), and ZR-75-1 (**I**,**J**) cells treated with different concentrations (25 μg/mL to 300 μg/mL) of rGO for 24 h (blue lines) and 48 h (red lines), and marked with asterisks. Percentage of LDH leakage or PI uptake was measured by LDH or PI test, respectively. The proliferation analysis of MDA-MB-231 (**K**) and ZR-75-1 (**L**) cells treated with different concentrations (25 μg/mL to 300 μg/mL) of rGO for 24 h (blue lines) and 48 h (red lines) using crystal violet assay. The mean values from three independent experiments ± SD are presented. Significant alterations are expressed relative to the controls of independent experiments ± SD are presented. Significant alterations are expressed relative to the controls and marked with asterisks. Statistical significance was considered if * *p* < 0.05, and marked with asterisks.

The cytotoxicity effect of rGO on T-47D, MCF-7, ZR-75-1, MDA-MB-231, Hs 578T cell lines (Figure 1A–J), was determined using LDH (Figure 1B,D,F,H,J) and PI (Figure 1A,C,E,G,I) tests. In our experiment, the cells were incubated with increasing concentrations of rGO (25–300 μg/mL), for 24 h (Figure 1, blue lines) and 48 h (Figure 1, red lines). It has been shown that rGO caused time-dependent and dose-dependent decrease in the cell viability only in MDA-MB-231 and ZR-75-1 cell lines, but not in T-47D, MCF-7, Hs 578T. The increase leakage of LDH and PI uptake in ZR-75-1 and MDA-MB-231 cells was observed after 24 h and 48 h of incubation with all rGO concentrations. Interestingly, in cells treated with higher concentrations of rGO, the effect of LDH leakage and PI uptake was more pronounced with the MDA-MB-231 line compared to the ZR-75-1 cell line. Prolongation of incubation time up to 48 h, in cells incubated with rGO, resulted in a strong leakage of LDH and PI uptake in ZR-75-1, MDA-MB-231 cells. Given this, based on the results of LDH leakage and PI uptake by MDA-MB-231 and ZR-75-1, rGO was selected for further studies at two concentrations, 50 μg/mL and 100 μg/mL.

The antiproliferative activity of rGO in MDA-MB-231 (Figure 1K) and ZR-75-1 cells (Figure 1L) was determined using crystal violet assay. In our experiment, the cells were incubated with increasing concentrations of rGO (25–300 μg/mL), for 24 h (Figure 1, blue lines) and 48 h (Figure 1, red lines). As shown in Figure 1K,L rGO markedly inhibited proliferation of MDA-MB-231 and ZR-75-1 cells. It has been demonstrated that rGO caused time-dependent and dose-dependent decrease in proliferation in MDA-MB-231 and ZR-75-1 cells.

### 2.2. The Effect of rGO on Oxidative Stress

**Figure 2:**
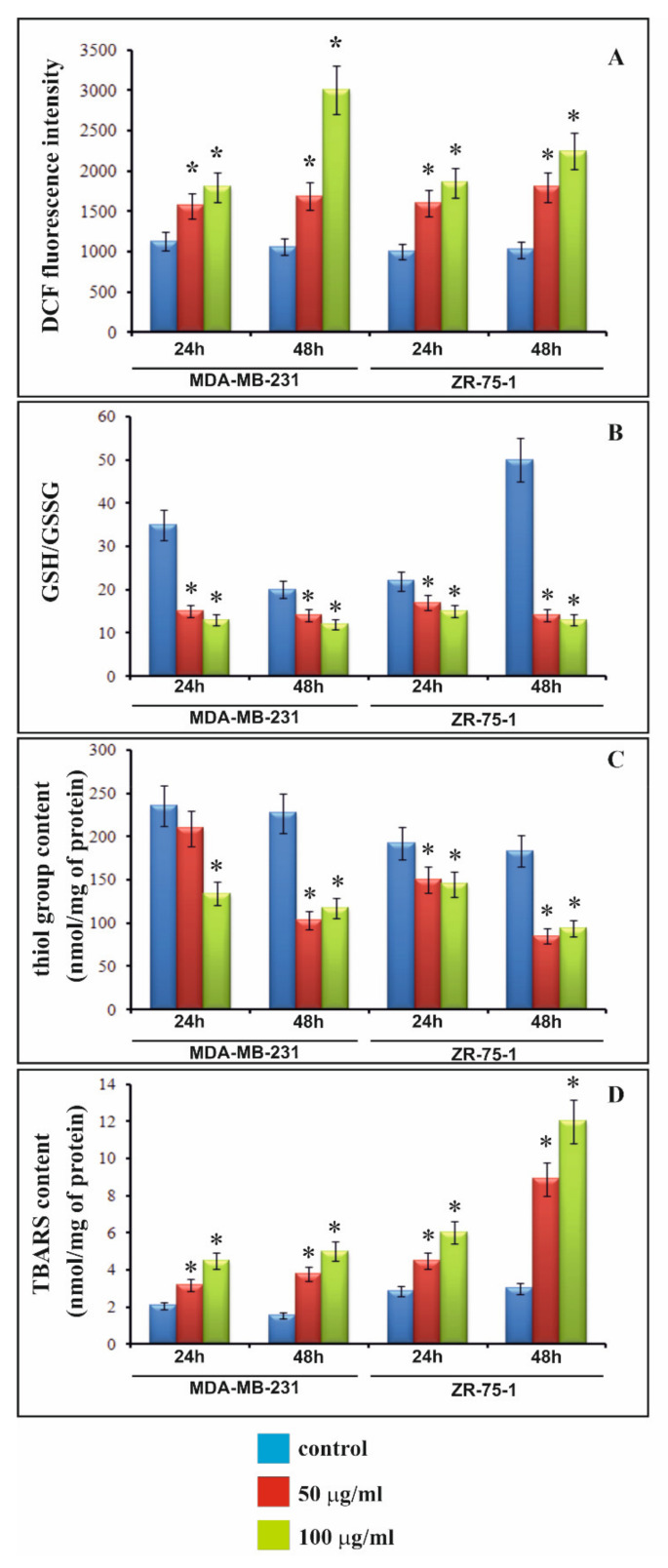
Oxidative stress and biochemical changes induced by rGO in MDA-MB-231 and ZR-75-1 cell lines. The cells were incubated with 50 μg/mL or 100 μg/mL rGO for 24 h and 48 h. Intracellular reactive oxygen species (ROS) production in MDA-MB-231 and ZR-75-1 cells is presented on panel (**A**). Panel (**B**) shows the GSH/GSSG ratio. The content of intracellular thiol groups is shown on panel (**C**), while panel (**D**) shows the TBARS content. Mean values from three independent experiments ± SD are presented. Significant alterations are expressed relative to the controls and marked with asterisks. Statistical significance was considered if * *p* < 0.05.

Figure 2A shows the relative fluorescence intensity of 2′7′-dichlorofluorescein (DCF) in MDA-MB-231 and ZR-75-1 cells exposed to 50 μg/mL or 100 μg/mL of rGO for 24 h and 48 h. The fluorescence of DCF was intensified with an increase in the intracellular ROS production and it was dependent on time of incubation and the concentration of rGO. After 24 h and 48 h incubation of MDA-MB-231 and ZR-75-1 cell lines with 50 μg/mL or 100 μg/mL of rGO, the intracellular ROS production was higher compared to the control cells (Figure 2). Figure 2B shows the GSH/GSSG ratio in MDA-MB-231 (left panel) and ZR-75-1 (right panel) cells incubated with 50 μg/mL or 100 μg/mL of rGO for 24 h and 48 h. After 24 h and 48 h incubation of MDA-MB-231 and ZR-75-1 cell lines with 50 μg/mL or 100 μg/mL of rGO, the GSH/GSSG ratio was lower in comparison to the control cells (Figure 2B). Figure 2C shows the content of thiol groups in MDA-MB-231 (left panel) and ZR-75-1 (right panel) cells incubated with 50 μg/mL or 100 μg/mL of rGO for 24 h and 48 h. After 24 h and 48 h incubating of MDA-MB-231 and ZR-75-1 T cell lines with 50 μg/mL or 100 μg/mL of rGO, the thiol groups content was lower compared to the control cells (Figure 2C). Figure 2D shows the TBARS content in investigated cells exposed to 50 μg/mL or 100 μg/mL of rGO for 24 h and 48 h. After 24 h and 48 h of incubation of the MDA-MB-231 and ZR-75-1 cell lines with 50 μg/mL or 100 μg/mL of rGO, the TBARS content was higher in comparison to the control cells (Figure 2D).

### 2.3. The Effect of rGO on Apoptosis and Necrosis

**Figure 3:**
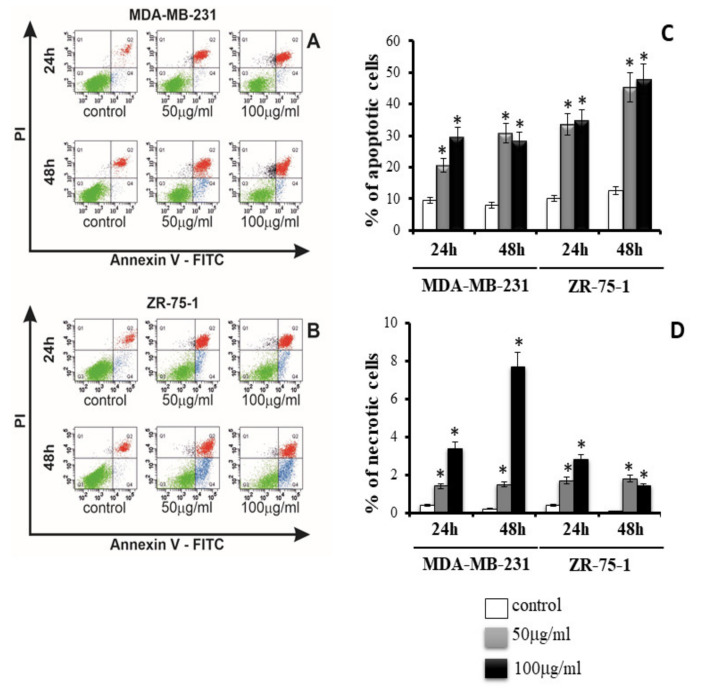
The effect of rGO on apoptosis (**A**–**C**) and necrosis (**A**,**B**,**D**) of MDA-MB-231 and ZR-75-1 cells was evaluated by Annexin V assay. The cells were incubated for 24 h and 48 h in medium with 50 μg/mL or 100 μg/mL rGO. The cells were double-stained with FITC-Annexin V and PI. Representative dot plots for Annexin V-FITC/PI staining are shown (**A**,**B**). The bar graphs present the percentage of apoptotic cells (**C**) and necrotic cells (**D**). Following acquisition of the sample, the cells were gated by the forward scatter FSC and side scatter SSC and analyzed for fluorescence intensity of FITC-Annexin V/PI. The cells were divided into four subpopulations: live cells-Q3 (Annexin V-FITC-/PI-), early apoptotic cells—Q4 (Annexin V-FITC+/PI-), late apoptotic cells-Q2 (Annexin V-FITC+/PI+), and necrotic cells—Q1 (Annexin V-FITC-/PI+). Percentage of apoptotic cells was the sum of percentage of early apoptotic (Q4) and late apoptotic cells (Q2). Mean values from three independent experiments ± SD are presented. Significant alterations are expressed relative to adequate controls and marked with asterisks. Statistical significance was considered if * *p* < 0.05.

The apoptosis and necrosis of MDA-MB-231 (Figure 3A,C,D) and ZR-75-1 (Figure 3B,C,D) cells were evaluated by flow cytometry on FACSCanto II (BD, San Diego, CA, USA). Figure 3C shows the percent of apoptotic cells in cultures incubated for 24 h and 48 h in medium with 50 μg/mL or 100 μg/mL rGO. Figure 3A shows representative histograms of MDA-MB-231, and ZR-75-1 cells (Figure 3B), FACS analysis by Annexin V-FITC and PI staining. As depicted in Figure 3C, we found a time- and dose-dependent increase in apoptosis in MDA-MB-231 and ZR-75-1 cells. After 24 h of incubation of MDA-MB-231 and ZR-75-1 cells treated with 50 μg/mL and 100 μg/mL of rGO, the percent of apoptotic cells was significantly higher in comparison to the control cells. Interestingly ZR-75-1 cells were more sensitive to rGO than MDA-MB-231 cells. As shown in Figure 3C, after 48 h of incubation in cells treated with 50 μg/mL and 100 μg/mL rGO, it increases apoptosis to about 30% (MDA-MB-231) and up to 40% (ZR-75-1). As depicted in Figure 3D, we found a time- and dose-dependent increase in necrosis of MDA-MB-231 and ZR-75-1. After 24 h and 48 h of incubation of MDA-MB-231 and ZR-75-1 cells treated with 50 μg/mL and 100 μg/mL rGO, the percent of necrotic cells was significantly higher in comparison to the control.

### 2.4. The Effect of rGO on Apoptosis (Fluorescence Method)

**Figure 4:**
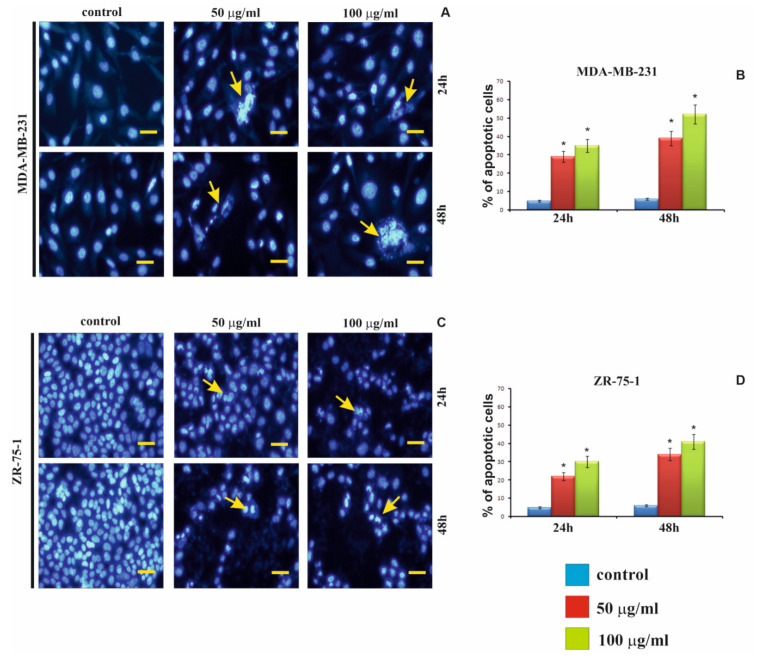
The effect of rGO on apoptosis in the MDA-MB-231 (**A**,**B**) and ZR-75-1 (**C**,**D**) cells was assessed by fluorescence microscopy. The cells were incubated in medium with rGO (50 μg/mL or 100 μg/mL) for 24 h and 48 h, next were stained with DAPI and photographed under a fluorescence microscope at 200-fold magnification and analyzed according to the following criteria: living cells-normal blue nucleus without chromatin condensation, apoptotic cells-blue stained nuclei with chromatin condensation, fragmentation or apoptotic body. We presented representative images from one of the three independent experiments. Scale bar 50 μm. The bar graphs present the percentage of apoptotic cells—(**B**,**D**). Significant alterations are expressed relative to controls and marked with asterisks. Statistical significance was considered if * *p* < 0.05.

Staining with fluorescence dye—DAPI was used for evaluation of the apoptotic cells morphology (Figure 4). Figure 4B,D shows the percent of apoptotic cells incubated in medium with rGO (50 μg/mL or 100 μg/mL) for 24 h and 48 h. Condensation and marginalization of chromatin or apoptotic body are known markers of apoptosis. The effect of rGO (50 μg/mL or 100 μg/mL) on apoptosis in MDA-MB-231 and ZR-75-1 cell lines was evaluated by fluorescence (DAPI staining), and the results were shown in Figure 4A,C while in Figure 4B,D shows the percentage of apoptotic cells. We observed that the MDA-MB-231 and ZR-75-1 cells incubated for 24 h and 48 h, with rGO (50 μg/mL or 100 μg/mL), caused chromatin accumulation, condensation and marginalization and the formation of apoptotic bodies (marked by yellow arrows). Furthermore, as depicted in Figure 4B,D, we found a time- and dose-dependent increase in percentage of apoptosis of MDA-MB-231 and ZR-75-1 cells, confirming the results of the flow cytometry analysis.

### 2.5. The Effect of rGO on Morphological Changes

**Figure 5:**
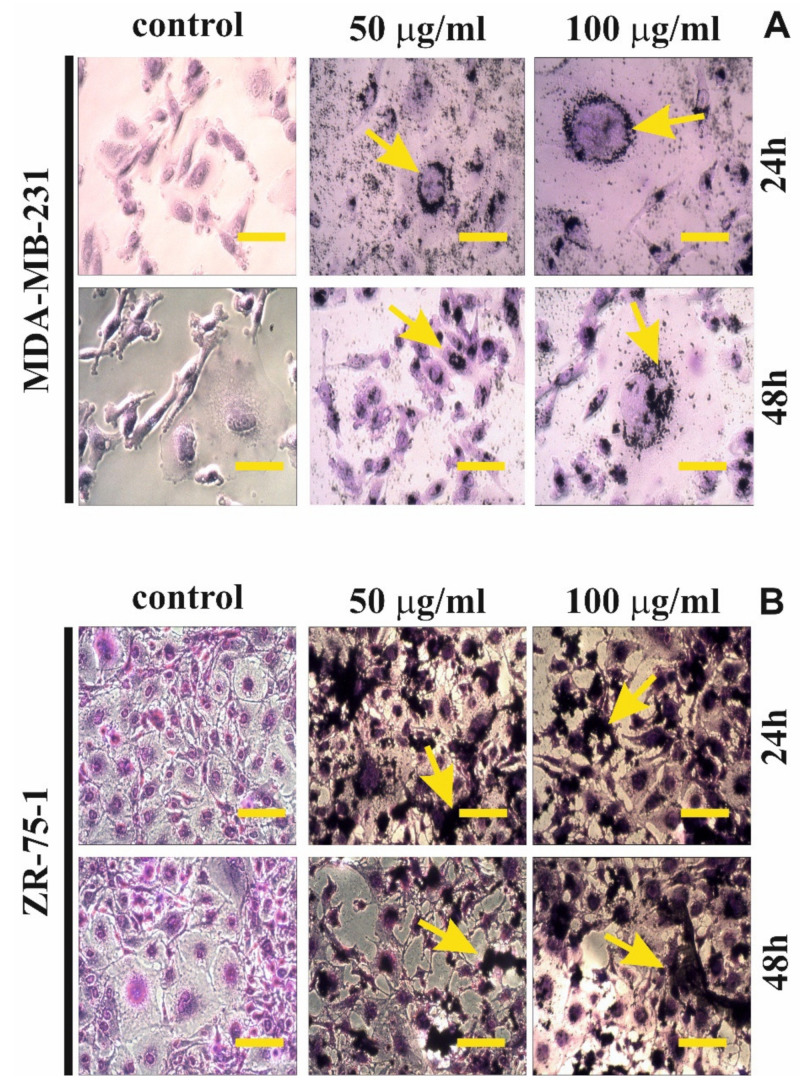
The effect of rGO on morphological changes of MDA-MB-231 (**A**) and ZR-75-1 (**B**) cells evaluated under inverted microscope. The cells were incubated for 24 h and 48 h in 50 μg/mL or 100 μg/mL rGO. The cells were observed and photographed under an inverted microscope at 200-fold magnification. We presented representative images from one of the three independent experiments. Scale bar 50 μm.

Figure 5 illustrates the morphological changes of the MDA-MB-231 and ZR-75-1 cells after exposure to 50 μg/mL and 100 μg/mL rGO, for 24 h and 48 h. The effect of rGO on the MDA-MB-231 and ZR-75-1 cells was examined under an inverted microscope. We noticed that, rGO were visible (black materials) and that the agglomerates adhered to the cell surface membrane. This observation was similar in cells that were incubated with rGO for 24 h and 48 h. Interestingly, we observed the accumulation of rGO around the nucleus. The morphological changes were more pronounced at 100 μg/mL than at 50 μg/mL and longer incubation times with rGO. In addition, the cells were more oval and shrunken compared to the control cells.

## 3. Discussion

Breast cancer growth is regulated by a set of interactions between hormones and growth factors with their specific cell receptors. These regulators may be produced by the cell itself or by surrounding cells. The estrogen receptor (ER) and the progesterone receptor (PR), similar to other members of the steroid hormone receptor family, are ligand-induced transcription factors that play an important role in the biology of the cancer mammary gland [17].

The MDA-MB-231 and ZR-75-1 cell lines are widely used in breast cancer modeling [18,19]. Ductal carcinoma is the most common histological category of breast cancer, and luminal A is the major molecular subtype. Interestingly, the differences can be characterized by the following property: ZR-75-1 cells with a luminal molecular subtype A have a morphology of mass aggregates [19]. Luminal breast cancer cells are characterized by positive ER and/or PR expression, despite the existence of some exceptional cases such as IBEP-1 and IBEP-3 where a positive PR influences their luminal phenotype. Moreover, luminal cells are comparatively more differentiated and less prone to migration due to tight cell-cell junctions, which is consistent with that at the tumor level [18]. MDA-MB-231 cells with a basic molecular subtype have a stellate aggregate morphology. In addition, MDA-MB-231 cells are ER, PR and E-cadherin negative and express mutant p53. In microarray profiling, the MDA-MB-231 cell genome is clustered with the primary breast cancer subtype. Since cells also lack the HER2 growth factor receptor, they provide a good model of triple negative breast cancer [19].

Molecular profiles and different morphology of MDA-MB-231 and ZR-75-1 cell lines may determine their different sensitivity to rGO. MDA-MB-231 and ZR-75-1 cells have either a mass aggregate morphology or a star aggregate morphology, respectively. This determines the significant contact area of cells with rGO and their sensitivity to rGO.

rGO is widely used as a drug or gene delivery system and its use in breast cancer therapy has been suggested [20]. Several studies have shown cytotoxic effects of GO on normal, cancerous and bacterial cells, although toxicological information on rGO materials remains limited [11,13,21]. In our study, we examined the cytotoxic effects of rGO nanomaterials on breast cancer cell lines.

Hydrophobic forms of graphene materials bind to the lipids of the cell membrane. It is interesting that the other form of graphene can affect cell receptors and disrupt metabolism by inhibiting glucose or amino acid supply, which leads to the induction of endoplasmic reticulum (ER) stress, apoptosis or autophagy. Moreover, graphene itself can bind intracellular nutrients from the cell culture medium, which limits their availability and inhibits cellular proliferation and viability [4,5].

GO is smaller and less toxic than rGO, which is believed to be due to its high oxygen content, smoother edges, and hydrophilic properties. rGO is characterized by a high affinity to the cell membrane, and irregular and sharp edges affect their integrity, stimulate receptors and activate mitochondrial pathways that can cause cell death [4,14].

Thus, the physical and chemical properties of rGO play a crucial role in graphene distribution and toxicity, leading to a significant reduction in cell viability [2,13]. The mechanisms of rGO cytotoxicity are not fully understood and depend on its physiochemical properties [14]. Analysis by transmission electron microscopy (TEM) showed that rGO has an irregular shape with sharp and jagged edges [9]. Jaworski et al. showed a strong tendency of graphene platelets to cluster close to the body of glioma cells [9]. Conversely, Akhavan et al. indicated that the irregular and sharp edges of GO may damage the integrity of the cell membrane [22]. It has been shown that rGO caused time-dependent and dose-dependent decrease in the cell viability in MDA-MB-231 and ZR-75-1 cell lines, but not in T-47D, MCF-7, Hs 578T. For this reason we choose MDA_MB-231 and ZR-75-1 cell lines for the next study. The differences in the sensitivity of Hs 578T, MCF-7, T47D breast cancer cell lines to rGO in comparison to the MDA-MB-231 and ZR-75-1 cells may result from differences in the expression of genes involved in apoptosis or autophagy. The rGO-treated cells were smaller in comparison to the control cells. Jaworski et al. showed graphene interacts with glioma cells and that GO is usually associated with cell bodies [9]. When the rGO sheet interacts with cells, the sharp edges of rGO can damage the cell membrane and, consequently, lead to the release of LDH [4]. Li et al. showed the redistribution of LDH, suggesting the significant loss of plasma membrane integrity. Similar results have also been observed in other cell types, including MCF-7 and Panc-1 cancer cells [21]. Data from the LDH assay and propidium iodide (PI) uptake showed that exposure to rGO resulted in a dose- and time-dependent decrease in cell viability, although this was only observed in estrogen-independent cells. Lammel et al. observed the effect of GO on the ultrastructure of the plasma membrane, while others have shown that nanosized GO penetrate the plasma membrane [23]. Gurunathan et al. have reported that GO decreases cell viability in a dose-dependent manner in estrogen receptor-positive MCF-7 cells [24]. Zhang et al. indicated that rGO exhibited higher toxicity than GO, possibly due to its hydrophilic properties, smoother edges, and high oxygen content of GO which reduce its potency [4]. Xu and coworkers demonstrated that GO can disrupt the integrity of the cell membrane by manipulating the expression of membrane- and cytoskeleton-associated genes. Furthermore, after internalization GO can be localized on F-actin filaments and thus induce cell cycle modifications, apoptosis or oxidative stress [25].

The main factor causing rGO-induced cytotoxicity is the production of ROS. Three main types of ROS are known: superoxide anion (O_2_^−^), hydroxyl radical (HO·) and hydrogen peroxide (H_2_O_2_), which play pivotal roles in cell metabolism, signalling and homeostasis [4]. However, ROS can induce the inactivation of intracellular proteins, lipid peroxidation, mitochondrial dysfunction, DNA damage and consequently lead to apoptosis or necrosis [4,26]. In vitro studies have shown that rGO causes oxidative stress, which disturbs the balance between the production of cellular ROS and the mechanisms of ROS detoxification [9]. Therefore, we decided to study the effect of rGO on intracellular ROS generation in MDA-MB-231 and ZR-75-1 cells. Our research showed that rGO induced intracellular ROS generation in both breast cancer cell lines in a dose- and time-dependent manner. Similarly, Zhang et al. showed that treatment with GO increases the level of ROS, decreases super oxide dismutase (SOD) activity, and induces MDA production in HeLa cells [24]. Sasidharan et al. suggested that carbon nanomaterials can induce intracellular ROS generation and this phenomenon consists in an imbalance between oxidant and antioxidant mechanisms [27]. Pulskamp et al. indicated that non-purified carbon nanotubes induce dose- and time-dependent ROS generation in rat macrophages and human lung cells [28]. We suggest that the strong increase in ROS production could lead to apoptosis of breast cancer cells. Fauser et al. indicated that apoptosis may results from reduced levels of the primary antioxidant GSH and can be connected with cell cycle disruptions, including G0/G1 reduction or S and M cell cycle arrest, together with a strong increase in ROS generation [29].

In the presented studies rGO enhanced the production of TBARS in breast cancer cells, especially in ZR-75-1 cells during a 48 h incubation. A significant increase in the amount of lipid peroxidation products amount is in accordance with the other results—a decrease in the content of thiol groups as well as an increase GSH/GSSG ratio and in an increase in ROS content under the influence of rGO. In order to assess the effect of studied compound on proteins, the study used one of the most popular markers of protein oxidation—the content of thiol groups. The obtained results confirmed that the tested compound shows pro-oxidative activity towards proteins and that this action is connected with its inhibitory effect on cells proliferation. rGO caused lipid bilayer and membrane protein damage by lipid peroxidation and oxidative damage to proteins [12]. Our observations are in agreement with the results of above mentioned studies, which indicate that the tested compound may induce oxidative stress. The free radicals generated under the influence of rGO can activate the caspase cell cascade and stimulate the apoptosis process. We noticed, that increased apoptosis was accompanied by a strong decrease in cell proliferation. Wang et al. indicated that GO inhibits cell proliferation and induces apoptotic cell death in glioblastoma stem-like cells (GSCs). Apart from that, GO can effectively inhibit the migration and invasion of human breast cancer cells, prostate cancer cells and mouse melanoma cells. It is interesting, that GO treatment may regulate the expression levels of cell cycle regulatory proteins and led to the cell cycle arrest [30].

Apoptosis and necrosis are two of the main types of cell death, which have been discussed in previous reports [6,9,10]. The two main mechanisms for inducing apoptosis include the mitochondrial (intrinsic) pathway and receptor-mediated (extrinsic) pathway. Interestingly, breast cancer cell lines are characterized by different activity of the proteins involved in apoptosis and cell cycle progression [1]. Our research showed that rGO induces apoptosis and necrosis in MDA-MB-231 and ZR-75-1 cells. Furthermore, Gurunathan et al. indicated that rGO induced apoptosis in human ovarian A2780 cancer cells by upregulation of caspase-3 [24]. It is interesting that we also observed increased apoptosis in MDA-MB-231 and ZR-75-1 cells. We also observed that when MDA-MB-231 and ZR-75-1 cells were incubated with rGO for 24 h and 48 h, they displayed morphological changes similar to those caused by apoptosis such as shrinkage, nuclear condensation, fragmentation, and the appearance of apoptotic bodies. The mechanism of necrosis is already well understood and consists in increased damage to the cell membrane. This, in turn, leads to rapid reduction of intracellular ATP levels and loss of osmotic balance of the cells, while apoptosis can be induced by strong oxidative stress. Li et al. indicated that GO caused a decrease in MMP and an increase in intracellular ROS generation, which then triggers apoptosis through the mitochondrial pathway [21]. Yang et al. indicated that GO can induce cytotoxicity, in macrophages, through the depletion of the MMPs, increasing the production of ROS, and can lead to apoptosis via the MAPK and TGF-β signaling pathways [21]. Furthermore, apoptosis can be caused by lipid peroxidation products. There is extensive literature on the biological effects of lipid peroxidation products, lipid hydroperoxides and aldehydes, which provides evidence that they can mediate cell death and decrease proliferation [12].

## 4. Materials and Methods

### 4.1. Reagents

The Dulbecco’s modified Eagle’s medium (DMEM), containing glucose at 4.5 mg/mL with GlutaMax^TM^, DPBS, trypsin-EDTA, medium RPMI, penicillin, streptomycin and fetal bovine serum Gold (FBS Gold) were provided by Gibco (San Diego, CA, USA). FITC Annexin V apoptosis detection Kit I was provided by BD Pharmingen^TM^ (San Diego, CA, USA). Reduced graphene oxide (rGO), paraformaldehyde, 3,8-Diamino-5-[3-(diethylmethylammonio)propyl]-6-phenylphenanthridinium diiodide-PI, 2′,7′-dichlorofluorescin diacetate-DCFH-DA,4′,6′-di-amidino-2-phenylindole-DAPI, and crystal violet by Sigma-Aldrich (St. Louis, MO, USA). SDS, TCA, TBA, Folin-Ciocalteu reagent were provided by Sigma-Aldrich (St. Louis, MO, USA). GSH/GSSG-Glo^TM^ Assay kit was provided by Promega (Madison). LDH-Cytotoxicity Assay kit (C) was provided by BioVision (CA, Milpitas, USA). Methanol, NaOH, and formaldehyde were provided by POCH (Gliwice, Poland).

### 4.2. Cell Cultures and Exposure to rGO

Human breast cancer cell lines: T-47D, MCF-7, ZR-75-1, which are estrogen receptor positive, and breast cancer cell lines: MDA-MB-231, Hs 578T, which are estrogen receptor negative, were obtained from American Type Culture Collection (ATCC). The MDA-MB-231, Hs 578T and MCF-7were cultured in DMEM, while the T-47D and ZR-75-1 were cultured in RPMI. The medium were supplemented with 10% heat-inactivated fetal bovine serum GOLD (FBS GOLD), penicillin (100 U/mL), and streptomycin (100 µg/mL). Cells were cultured in Falcon flasks (BD Pharmingen^TM^, San Diego, CA, USA) in CO_2_ incubator Galaxy S+ (RS Biotech, Irvine, UK), at 37 °C, 5% CO_2_ and 95% air in an incubator Galaxy S+ (RS Biotech, Irvine, UK). At approximately 70% confluence, cells were detached with 0.05% trypsin, 0.02% EDTA and counted in a Scepter Cell Counter (Millipore, MA, USA). Subsequently, known number of cells, (2.5 × 10^5^ per well) were seeded in 2 mL of DMEM or RPMI in six-well plates. In order to minimize the aggregation of rGO, prior to the experiments nanoparticles were dispersed in deionized water (1 mg/mL) by a sonicator (Sonopuls, Bandelin, Berlin, Germany), on ice for 45 min (160 W, 20 kHz). Next, after 24 h incubation, DMEM or RPMI were removed and replaced with fresh DMEM or RPMI containing rGO suspensions, at concentrations ranging from 25 μg/mL to 300 μg/mL. The cell lines not treated with rGO served as the negative controls. After, the cells were incubated for 24 h and 48 h and retained for further analyses.

### 4.3. Propidium Iodide Uptake Assay

Viability of breast cancer cell lines on treatment with different concentration of rGO was assessed using propidium iodide (PI) staining. The measurement is based on the principle that PI is a membrane impenetrable dye that is generally excluded from live cells. It binds to double stranded DNA by intercalating between base pairs. PI is excited at 488 nm and emits at a maximum wavelength of 617 nm. The breast cancer cells (2.5 × 10^5^ per well) were seeded in 1 mL of medium in 6-well culture plate and allowed to adhere for 24 h at 37 °C. Next, the cells were treated with various, at 25, 50, 100, 200 and 300 μg/mL, concentrations of rGO. Cell lines were incubated for 24 h and 48 h. The cells were detached using trypsin-EDTA solution, resuspended in medium, then in DPBS and incubated with PI dye (50 μg/mL in DPBS) in dark, at room temperature, for 15 min. Subsequently, the cell suspension was diluted by adding 300 μL DPBS and proportions of live/dead cells were analyzed using flow cytometer, FACSDiva software (BD PharmingenTM, San Diego, CA, USA), and 10,000 cells were measured per sample. Dead cells are typically positive for PI, whereas live cells display PI negative.

### 4.4. Membrane Integrity

A lactic dehydrogenase (LDH) test was used to evaluate cells membrane integrity. The breast cancer cell lines were plated in 96-well plates (1 × 10^4^ cells per well) in 200 μL of medium and incubated for 24 h. After 24 h, the medium was removed, replaced with the rGO suspension in medium, at 25, 50, 100, 200 and 300 μg/mL concentrations and incubated for 24 h and 48 h. A total of 100 μL of the lactate dehydrogenase assay mixture was added to each well. The culture was covered and incubated for 20 min at RT. The OD was recorded as outlined and the LDH leakage was expressed as the percentage of OD. If the cell membrane is damaged, intracellular LDH molecules are released into the culture medium. The LDH level in the medium indicates cell membrane damage.

### 4.5. Proliferation Test

The MDA-MB-231 and ZR-75-1 cells were seeded (0.5 × 10^5^ per well) in 1 mL of medium in 24-well culture plate and allowed to adhere for 24 h at 37 °C. Next, the cells were treated with various, at 25, 50, 100, 200 and 300 μg/mL, concentrations of rGO. Both cell lines were incubated for 24 h and 48 h. Subsequently, they were washed with cold DPBS and then fixed in 1% formaldehyde in DPBS for 10 min at room temperature. After fixation, the cells were permeabilized in 1% methanol for 5 min and the cells were stained with a mixture of 0.05% crystal violet. Next, the intracellular crystal violet product was dissolved in methanol, and absorbance was measured at λ = 590 nm in a microplate reader (Tecan, Männedorf, Switzerland). The proliferation of rGO-treated breast cancer cells was calculated as a percentage of control untreated cells. All the experiments were conducted in duplicate in at least three cultures.

### 4.6. Intracellular ROS Detection

The level of intracellular ROS was determined using dichlorodihydrofluorescein diacetate (DCFH-DA) assay, (Sigma, St. Louis, MO, USA). After diffusion through the cell membrane, DCFH-DA is deacetylated by cellular esterases to a non-fluorescent compound, which is later oxidized by intracellular ROS into a fluorescent 2′,7′-dichlorofluorescein (DCF). The MDA-MB-231 and ZR-75-1 cells (1 × 10^4^ cells per well) were seeded in 200 μL of DMEM or RPMI in 96-well black plates. After 24 h, DMEM or RPMI was removed and the cells were stained with 10 μM of DCFH-DA in PBS at 37 °C, 5% CO_2_, for 45 min. Then, the dye was removed and replaced with the rGO suspensions in DMEM or RPMI, at 50 μg/mL or 100 μg/mL concentrations and incubated for 24 h and 48 h. The DCF fluorescence intensity was measured by Infinite M200 microplate reader (Tecan, Männedorf, Switzerland), at the excitation wavelength of 485 nm and the emission wavelength of 535 nm. The intracellular ROS generation in rGO-stimulated breast cancer cells was shown as the intensity of fluorescence of the DCF.

### 4.7. Determination of SH Groups

SH-groups were measured using the method of Rice-Evans [31], as described previously by Jabłońska-Trypuć et al. [32]. The MDA-MB-231 and ZR-75-1 cells (2.5 × 10^5^ cells/mL) were incubated in 2 mL of medium with or without the test rGO (50 μg/mL or 100 μg/mL) in tissue culture 6-well plates. 

### 4.8. Determination of TBA Reactive Species (TBARS) Levels

The level of membrane lipid-peroxidation products, or TBARS, was measured using the method of Rice-Evans [32], as described previously by Jabłońska-Trypuć et al. [31]. The MDA-MB-231 and ZR-75-1 cells (2.5 × 10^5^ cells/mL) were incubated in 2 mL of medium with or without the test rGO (50 μg/mL or 100 μg/mL) in tissue culture 6-well plates. 

### 4.9. Determination of GSH/GSSG

Total glutathione and GSH/GSSG ratio were each assayed in triplicate via GSH/GSSG-Glo™ kit (Promega Madison, Fitchburg, WI, USA) following manufacturer’s instructions. The MDA-MB-231 and ZR-75-1 cells were seeded in white bottom 96-well plates at 10^4^ cells/well (Sarstedt), allowed to attach, and treated with rGO (50 μg/mL or 100 μg/mL). Prior to the assay growth media were removed and cells washed with PBS. Assay is based on a luminescence measurement and detects and quantifies total glutathione (GSH + GSSG), GSSG and GSH/GSSG ratios in cultured cells. Stable luminescent signals are correlated with either the GSH or GSSG concentration of a sample. In this method GSH-dependent conversion of a GSH probe, Luciferin-NT, to luciferin by a glutathione S-transferase enzyme is coupled to a firefly luciferase reaction. Light from luciferase depends on the amount of luciferin formed, which in turn depends on the amount of GSH present. Thus, the luminescent signal is proportional to the amount of GSH. GSH/GSSG ratios are calculated directly from luminescence measurements.

### 4.10. Detection of Apoptosis and Necrosis

Apoptosis and necrosis of breast cancer cell lines were evaluated by flow cytometry on FACSCanto II cytometer (BD, San Diego, CA, USA). The MDA-MB-231 and ZR-75-1 cells (2.0 × 10^5^ per well) were seeded in 2 mL of medium in six-well plates. After 24 h, the medium was removed, replaced with the rGO suspension in medium, at 50 μg/mL or 100 μg/mL concentrations. All cell lines were incubated for 24 h and 48 h. The cells were detached, resuspended in medium and then in binding buffer. Subsequently, the cells were stained with FITC Annexin V and PI (FITC Annexin V apoptosis detection Kit I, (BD Pharmingen^TM^, San Diego, CA, USA) at room temperature, in the dark, for 15 min. Data were analyzed using FACSDiva software (BD PharmingenTM, San Diego, CA, USA). 

### 4.11. DAPI Staining

The MDA-MB-231 and ZR-75-1 cells were seeded (0.5 × 10^5^ per well) in 1 mL of medium in 24-well culture plate and allowed to adhere for 24 h at 37 °C. Next, the cells were treated with various, 50 μg/mL and 100 μg/mL, concentrations of rGO. Cell lines were incubated for 24 h and 48 h. After these times, they were washed with cold DPBS and then fixed in 4% paraformaldehyde in DPBS for 10 min at room temperature. After fixation, the cells were permeabilized in DPBS containing 0.2% Triton-X100 for 5 min. Then, the cells were washed three times in DPBS and stained with 0.01% DAPI for 10 min to indicate the nucleus. Samples were washed twice in DPBS and mounted on microscopy slides in medium cover-quick and stored in the dark until viewing. The cells were analyzed by using camera and a fluorescence microscope Olympus CXK41, U-RLFT50, equipped with filters DAPI (blue), (excitation wavelength/emission filter: 405/450 nm) at 200-fold magnification. The hundred cells per sample were analyzed by fluorescence microscope according to the following criteria: living cells-normal blue nucleus without of chromatin condensation; apoptotic cells-blue stained nuclei with chromatin condensation, fragmentation or apoptotic bodies.

### 4.12. Cell Morphological Analysis

To visualize morphological changes cell lines were exposed to rGO treatment and then stained with 1% crystal violet. Staining was followed by phase contrast microscopic observation. The MDA-MB-231 and ZR-75-1 cells, at a density of 2.0 × 10^5^, were seeded into 6-well plates and incubated with 50 μg/mL or 100 μg/mL of rGO at 37 °C in a humidified atmosphere containing 5% CO_2_ for 24 h and 48 h. Subsequently, they were washed with cold PBS and then fixed in 1% formaldehyde in DPBS for 10 min at room temperature. After fixation, the cells were permeabilized in 1% methanol for 5 min and the cells were stained with a mixture of 0.05% crystal violet. Next, the cells were washed two times with water and visualized using a phase contrast microscope Olympus, CKX 41 (Tokyo, Japan) at 200× magnification.

### 4.13. Total Protein Content in Cells

The MDA-MB-231 and ZR-75-1 cells (2.5 × 10^5^ cells/mL) were incubated in 2 mL of medium with/or without the test compound in tissue culture 6-well plates. Next, the cells were homogenization and extraction in 0.1 M NaOH at 4 °C total protein content was calculated. The concentration of proteins was determined spectrophotometrically as per Lowry et al. The absorbance of the extracts was measured spectrophotometrically at 750 nm [33].

### 4.14. Statistical Analysis

Mean values from three independent experiments ± standard deviations (SD) were calculated. The data were statistically analyzed using one way-ANOVA followed by Tukey’s post hoc *t*-test analysis. The significant differences of means were determined at the level of * *p* < 0.05.

## 5. Conclusions

**Figure 6:**
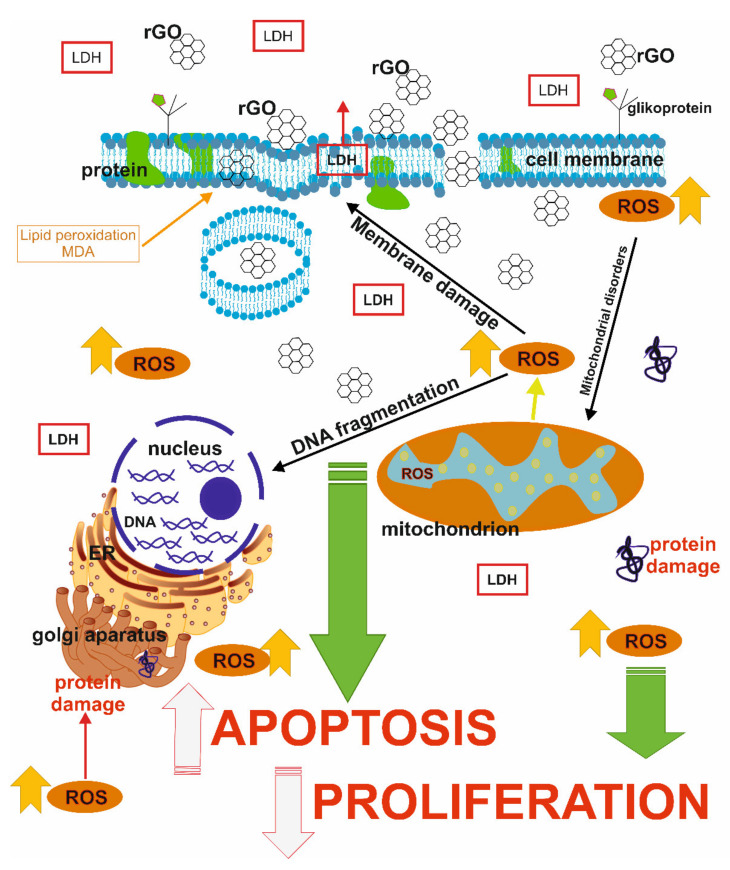
The effect of rGO on cytotoxicity, oxidative stress, apoptosis and proliferation in breast cancer cells. Abbreviation: rGO—reduced graphene oxide; ROS—reactive oxygen species; LDH—lactate dehydrogenase.

In conclusion, we suggest, that rGO can induce cytotoxicity in MDA-MB-231 and ZR-75-1 breast cancer cell lines, but not in T-47D, MCF-7, Hs 578T. We observed these two cell lines exhibit increased oxidative stress, up regulation of apoptosis, accumulation of nuclear condensation and marginalisation of chromatin. Furthermore, we indicated that rGO may reduce cell proliferation. These results may indicate that rGO is a promising therapeutic option in the fight against breast cancer cells (Figure 6).

## Data Availability

Not applicable.

## Supplementary Materials

The following are available online at https://www.mdpi.com/article/10.3390/ijms222212593/s1.
