# Supplementary figures and images for: The Preliminary Study on the Proapoptotic Effect of Reduced Graphene Oxide in Breast Cancer Cell Lines
Source: Int J Mol Sci. 2021 Nov 22;22(22):12593. doi: 10.3390/ijms222212593 (PMC8620501; doi:10.3390/ijms222212593)

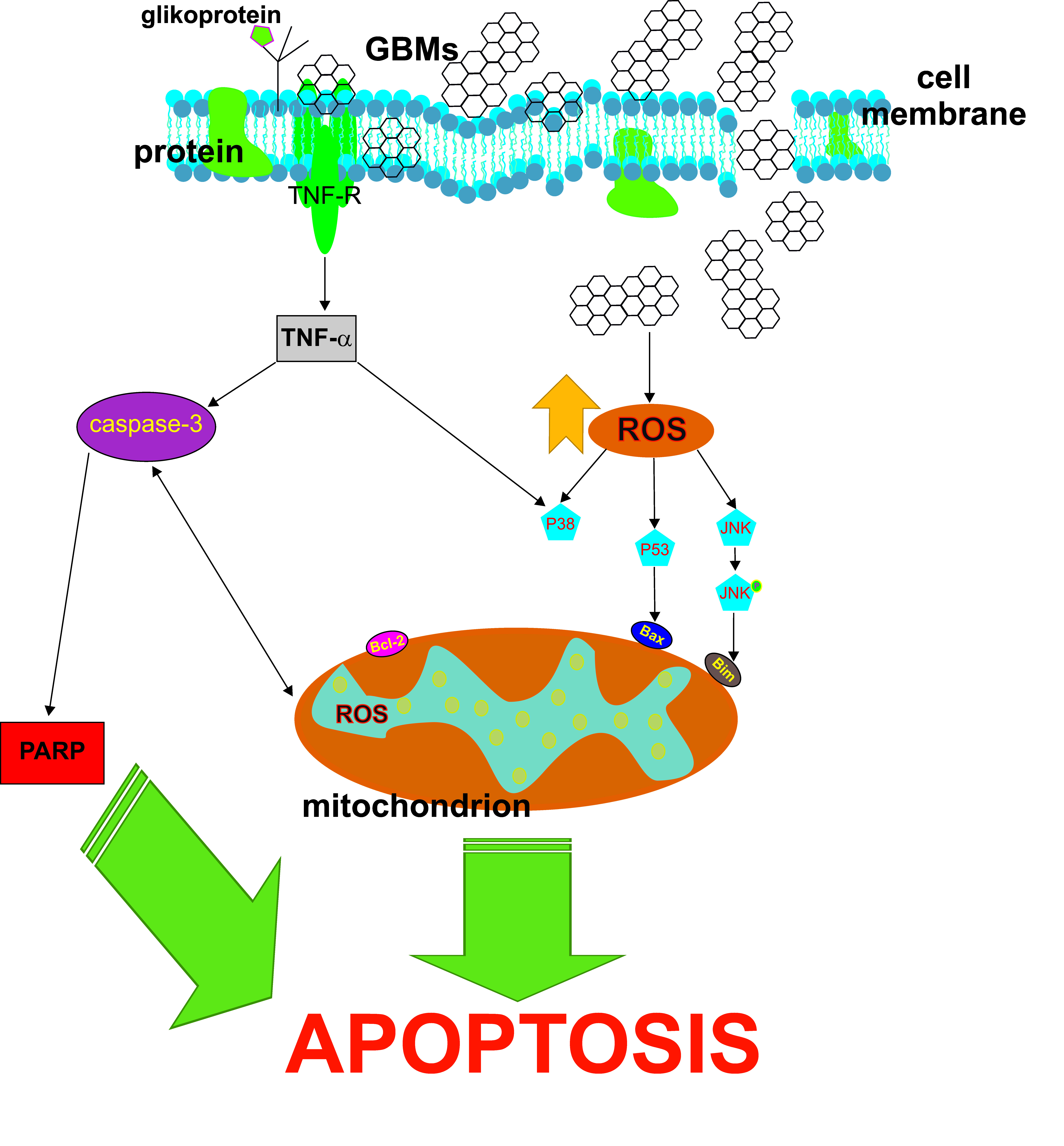

Supplement: Supplementary file 1 [file ijms-22-12593-s001.zip › ijms-1462486-supplementary.jpg]
